# Clinical Trial: Efficacy and Safety of Velusetrag in Chronic Intestinal Pseudo‐Obstruction: A Randomized, Phase 2, Placebo‐Controlled, Crossover, Multiple (*n* = 1), Proof‐of‐Concept Study

**DOI:** 10.1111/nmo.70246

**Published:** 2026-01-19

**Authors:** Carolina Malagelada, Roberto De Giorgio, Rosanna Francesca Cogliandro, Luis Alcalá‐González, Anna Costanzini, Valeria Scuderi, Sara Manzoni, Elena Pasquali, Jan Tack, Vincenzo Stanghellini

**Affiliations:** ^1^ Digestive System Research Unit Vall d'Hebron University Hospital Barcelona Spain; ^2^ Network Biomedical Research Center for Liver and Digestive Diseases (CIBEREHD) Madrid Spain; ^3^ Autonomous University of Barcelona Barcelona Spain; ^4^ Department of Translational Medicine University of Ferrara Ferrara Italy; ^5^ Internal Medicine Unit St. Annunziata Hospital Cento Ferrara Italy; ^6^ Internal Medicine and Digestive Pathophysiology Sant'Orsola‐Malpighi University Hospital Bologna Italy; ^7^ R&D Department Alfasigma S.p.A. Bologna Italy; ^8^ Department of Gastroenterology and Hepatology University Hospitals Leuven Leuven Belgium; ^9^ Department of Medical and Surgical Science University of Bologna Bologna Italy

**Keywords:** functional GI diseases, motility, small intestine

## Abstract

**Background:**

Chronic intestinal pseudo‐obstruction (CIPO) is a rare, severe disorder of gastrointestinal motility. Although 5‐hydroxytryptamine type 4 (5‐HT_4_) receptor agonists stimulate gastrointestinal motility, few trials have assessed their therapeutic effects in patients with CIPO.

**Aim:**

To assess the efficacy and safety of velusetrag, a highly selective 5‐HT_4_ receptor agonist, in patients with CIPO.

**Methods:**

This was a phase 2, placebo‐controlled, crossover, multiple (*n* = 1), multicenter, double‐blind, proof‐of‐concept trial. Eligible patients were aged 18–80 years, had CIPO (idiopathic or secondary to neurodegenerative disorders) and received ≥ 30% of their daily caloric intake orally. Over four periods, each of four weeks, patients were randomly assigned to once‐daily, oral velusetrag 15 mg (two periods) or placebo (two periods), with a 2‐week washout between each treatment period. The primary endpoint was the improvement in the weekly global gastrointestinal symptoms average index score (WGGSAIS) from the start to the end of each treatment period, assessed among patients who were responders or naive to previous 5‐HT_4_ receptor agonist treatment.

**Results:**

Overall, 17 patients with idiopathic CIPO received treatment and were included in safety analyses; efficacy was assessed in 15 patients. Mean (standard deviation) changes in WGGSAIS during treatment were −0.42 (0.693) for velusetrag and −0.19 (0.688) for placebo (between‐treatment difference: −0.24; 95% confidence interval: −0.553, 0.074; *p* = 0.1279). No deaths or serious or treatment‐related treatment‐emergent adverse events were reported.

**Conclusion:**

Velusetrag treatment was associated with improved symptoms versus placebo, although differences were not statistically significant at this sample size. Velusetrag was generally well tolerated.

**Trial Registration:**

ClinicalTrials.gov identifier: NCT05724069; EudraCT number: 2021‐000854‐24

## Key Point

- Velusetrag is a highly selective 5‐HT4 receptor agonist that has been shown to stimulate gastrointestinal motility.
- This phase 2 proof‐of‐concept trial evaluated the efficacy and safety of velusetrag in patients with chronic intestinal pseudo‐obstruction.
- Velusetrag treatment was generally well tolerated and was associated with improved symptoms and a reduction in pseudo‐obstructive episodes versus placebo, although these differences did not reach statistical significance in the small population involved in the study.

## Graphical abstract

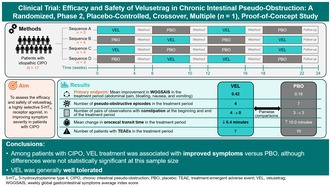

## 1 Introduction

Chronic intestinal pseudo‐obstruction (CIPO) is a rare disease characterized by severe impairment of intestinal propulsive activity, with symptoms resembling mechanical intestinal obstruction but in the absence of any physical occluding lesion [1, 2]. CIPO can be primary, idiopathic, or secondary to other conditions, such as neuromuscular or neurodegenerative diseases [1, 2, 3]. In a survey conducted in Japan, incidences of adult‐onset CIPO were estimated to be 0.21 and 0.24 cases per 100,000 men and women per year, respectively, with mean ages at diagnosis of 63.1 years (men) and 59.2 years (women) [4]. A pediatric intestinal pseudo‐obstruction retrospective cohort study conducted in the Netherlands estimated the incidence of pediatric CIPO to be 0.008 cases per 100,000 children per year [5].

CIPO has a heterogeneous presentation, but most patients have abdominal distension and/or pain, and associated symptoms can include nausea and vomiting, constipation, diarrhea, and urinary symptoms [6, 7]. Patients with CIPO typically show clinical and radiological features that mimic episodic or recurrent chronic mechanical obstruction of the intestine, such as bowel dilatation and air–fluid levels, but in the absence of an organic occlusion of the gut lumen [1]. CIPO is considered the most severe expression of disordered gastrointestinal motility [1]. Patients face long‐term severe digestive symptoms, challenges with maintaining optimal nutrition, limited knowledge and awareness of CIPO among healthcare practitioners, and poor efficacy of current medical treatments. Together, these lead to poor health‐related quality of life, life‐threatening complications, and, in some cases, repeated unnecessary surgical procedures [6, 8, 9, 10]. It is therefore important to distinguish CIPO from other conditions such as enteric dysmotility, which also presents with severe impairment of intestinal motility but without clinical and radiological features suggestive of intestinal obstruction [1].

In a survey of 154 expert clinicians conducted in 2018, 95% of respondents reported that they found it challenging to diagnose CIPO and enteric dysmotility, mainly owing to nonspecific symptoms [11]. In the same year, a European Society for Paediatric Gastroenterology Hepatology and Nutrition (ESPGHAN)‐led expert group developed consensus‐based recommendations for pediatric intestinal pseudo‐obstruction, covering topics ranging from diagnosis to management and treatment options [12]. Such recommendations are yet to be developed for adult‐onset CIPO, although consensus guidelines are now starting to emerge [13]. Indeed, it may take several years for patients to receive a proper diagnosis [1, 9, 11].

Treatment and management strategies for CIPO include prokinetics (such as 5‐hydroxytryptamine type 4 [5‐HT_4_] receptor agonists) to increase gastrointestinal motility, as well as antibiotics for small intestinal bacterial overgrowth (SIBO), nonopioid analgesics for intractable visceral pain, and parenteral or enteral feeding to counteract malnutrition [14, 15, 16]. Serotonin is a key molecule in gut–brain signaling and regulates functions such as gastrointestinal secretion, sensation, and motility [14]. Agonism of the 5‐HT_4_ receptor, one of seven main serotonin receptor subtypes, has been shown to stimulate intestinal propulsive motility [14] throughout the entire gastrointestinal tract, both in healthy individuals and in those with disorders of impaired gastrointestinal motility [17, 18]. Although prokinetics have a key role in the pharmacological management of CIPO [14, 19], there are no specifically approved agents, and few studies have assessed the effects of 5‐HT_4_ receptor agonism in patients with CIPO [20, 21].

Velusetrag is a potent, highly selective, pan‐gastrointestinal 5‐HT_4_ receptor agonist [22], shown to increase prokinetic gastrointestinal activity in several studies [23, 24, 25, 26], but its effects are yet to be demonstrated in CIPO. This phase 2, proof‐of‐concept trial investigated the efficacy and safety of velusetrag in improving symptom severity in patients with idiopathic CIPO.

## 2 Methods

### 2.1 Study Design

This was a phase 2, placebo‐controlled, crossover, multiple (*n* = 1), multicenter, double‐blind, proof‐of‐concept trial (NCT05724069, EudraCT 2021‐000854‐24). Patient recruitment was planned to take place in four hospitals located in Belgium, Italy (two sites), and Spain. Male and female patients were treated for four periods of 4 weeks each with oral, once‐daily velusetrag 15 mg (two periods) and placebo (two periods); there was a washout period of 2 weeks between each treatment period.

The study was approved by the independent ethics committee or institutional review board at the coordinating investigator's site (Comitato Etico Area Vasta Emilia Centro [CE—AVEC] at University of Bologna, IRCCS S. Orsola [no. 03901], reference number 652/2021/Farm/AOUBo) and at each individual study site (CE—AVEC at Arcispedale Sant'Anna di Ferrara [no. 03902], reference number 677/2021/Farm/AOUFe; Ethics Committee of the Hospital General Universitario Gregorio Marañón at Vall d'Hebron University Hospital [no. 03401], reference number 2021‐000854‐24; and Ethics Committee Research at UZ Leuven [no. 03201], reference number S65763). The study was conducted in accordance with the ethical and scientific principles governing clinical research as set out in the Declaration of Helsinki, the guidelines on Good Clinical Practice, and applicable national and local laws and regulations. All participants provided written informed consent before participating in the study.

### 2.2 Patients

Eligible patients were male or female, 18–80 years of age, had a diagnosis of idiopathic CIPO or CIPO secondary to primary neurodegenerative disorders, and received at least 30% of their daily caloric intake from oral nutrition. As no agreed diagnostic criteria exist for CIPO, diagnoses were confirmed by the principal investigator at each site and were based on a combination of clinical and radiographic findings, including small bowel manometry to confirm motor abnormalities, radiographic imaging to confirm bowel dilatation with air–fluid levels, and molecular and genetic testing [1]. Exclusion criteria included patients with primary CIPO (defined as congenital CIPO resulting from a genetic mutation [1]), patients with CIPO secondary to other known endocrine/metabolic and autoimmune diseases or CIPO secondary to neurologic conditions other than a neurodegenerative or demyelinating disease, and patients who were receiving total non‐oral nutrition. Detailed eligibility criteria are listed in Table S1.

### 2.3 Procedures

After a screening period of up to 7 days, eligible patients were randomly allocated (1:1:1:1) to one of four treatment sequences, each consisting of a total of four individual 4‐week treatment periods (and two complete treatment cycles) (Figure S1). The four treatment sequences were velusetrag–placebo–velusetrag–placebo, placebo–velusetrag–placebo–velusetrag, velusetrag–placebo–placebo–velusetrag, and placebo–velusetrag–velusetrag–placebo. Block randomization was conducted by a centralized randomization interactive web response system, and the assigned medication pack (either velusetrag or matching placebo) was dispensed to each patient at the start of each of the four treatment periods, recording the patient's assigned kit number. The study sponsor, contract research association, researchers, site staff, and patients were blinded to treatment allocation, and the medication packaging did not contain any information that could potentially unblind the patients and investigators to the treatment. Patients were stratified by 5‐HT_4_ receptor agonist responder status, as determined by the investigator. A non‐responder was defined as a patient with a history of a lack of benefit from 5‐HT_4_ receptor agonists, a responder was defined as a patient with a history of benefit from 5‐HT_4_ receptor agonists, and a naive patient was defined as a patient who had not previously received 5‐HT_4_ agonists (including prucalopride, cisapride, clebopride, and cinitapride).

### 2.4 Study Endpoints

The primary endpoint was the improvement in the weekly global gastrointestinal symptoms average index score (WGGSAIS) from the start to the end of each treatment period. The WGGSAIS of a patient was calculated as the mean of their scores for each of the four symptoms assessed, comprising abdominal pain, bloating, nausea, and vomiting. Symptoms were rated by the patient in an electronic diary for a recall period of 7 days on a Likert scale with the following categories: 0, absent; 1, mild (not influencing usual activities); 2, moderate (diverting from, but not urging modification of, usual activities); 3, severe (influencing usual activities markedly enough to urge modifications); and 4, extremely severe (precluding daily activities). A decrease in the WGGSAIS indicates an improvement in symptom severity.

In addition, a sensitivity analysis of the primary endpoint was performed using imputed values for missing data. If a pretreatment value was missing for a treatment period (whether with velusetrag or placebo), the patient's mean values obtained during their placebo treatment periods were used. If an end‐of‐treatment value was missing and intermediate values within a treatment period were available (from pretreatment up to the end of a treatment period), the last observation that occurred in that treatment period was used to impute the end‐of‐treatment value.

The following secondary endpoints were assessed: (1) the proportion of patients with at least a 1‐point improvement in the WGGSAIS from the start to the end of each treatment period; (2) the number of pseudo‐obstructive episodes from pretreatment to the end of each treatment period (self‐reported by patients and reviewed by the investigator at each visit); (3) the number of CIPO‐related hospitalizations during the treatment periods; (4) the change in stool consistency from pretreatment to the end of each treatment period, recorded by patients in an electronic diary and assessed using the Bristol stool scale (constipation, types 1 and 2; normal, types 3 and 4; and diarrhea, types 5, 6, and 7) [27]; and (5) the change in orocecal transit time from pretreatment to the end of the first treatment period. Orocecal transit time was measured using the lactulose breath test [28] at baseline (after a 12‐h fast) and at 15‐min intervals for 4 h after ingestion of a solution containing 100 mL water and 10 g lactulose. The lactulose breath test was performed during screening and also at the end of the first treatment period. Orocecal transit time was defined as the duration between ingesting lactulose and the first sustained rise of breath hydrogen, defined as an increase of at least 10 ppm from baseline that was maintained or increased in the two subsequent measurements. The study protocol did not specify a fixed period of antibiotic interruption, and its occurrence could bias the breath test results. Thus, an additional sensitivity analysis for orocecal transit time was conducted that excluded patients who took antibiotics in the 14 days preceding the lactulose breath test.

Safety and tolerability assessments included vital signs, an electrocardiogram, physical examinations, laboratory assessments, and documentation of treatment‐emergent adverse events (TEAEs). TEAEs were defined as adverse events starting on or after the first intake of treatment (i.e., the first intake in period 1). TEAEs were classified as velusetrag‐emergent if the last treatment taken before the TEAE onset date was velusetrag, or as placebo‐emergent if the last treatment taken before the TEAE onset date was placebo. In addition, treatment‐related TEAEs (TEAEs for which there is a reasonable possibility of a causal relationship between the event and the study treatment), serious TEAEs (TEAEs that were life‐threatening or that resulted in death, inpatient hospitalization, prolongation of existing hospitalization, persistent or significant disability/incapacity, congenital anomaly, or other medically important events), and deaths were documented.

### 2.5 Statistical Analyses

Sample size calculations were based on *t*‐test assessments of the primary endpoint analysis, which assessed the differences between velusetrag and placebo treatment periods within each paired treatment cycle (in the whole treatment sequence, there were up to two observable pairs per patient). A target of 16 patients was set, for a potential maximum of 32 pairs of evaluations. Accounting for 25% dropouts/missing pairs and a two‐sided significance level of 5%, 24 pairs would lead to power levels above 80% to detect an effect size of more than 0.7. A pair of observations was defined as 4 weeks of velusetrag or placebo followed by a 2‐week washout period, then 4 weeks of placebo or velusetrag (whichever the patient did not receive in the first treatment period) followed by a 2‐week washout period, both occurring within the same treatment cycle. Analysis was performed on evaluable pairs (i.e., evaluation available both for velusetrag and placebo within each cycle). Each patient could be evaluated twice, once for each cycle; thus, each patient could contribute 0, 1, or 2 observations for each treatment cycle.

Efficacy analyses were performed on the modified Full Analysis Set 1 (mFAS1) population, which included all randomized and treated patients who were either responders or naive to previous treatment with a 5‐HT_4_ receptor agonist and who had at least one valid measurement for the primary endpoint during a velusetrag treatment period and a placebo treatment period within the same cycle. Safety analyses were performed on the safety analysis set population, which included all patients who received at least one dose of velusetrag or placebo.

A stratified paired *t*‐test was used to analyze the treatment effect for the primary endpoint, with no data imputation for missing data pairs. Missing pairs (treatment effect difference for velusetrag minus placebo) could occur when at least one pretreatment or end‐of‐treatment value was missing for either velusetrag or placebo. For the proportion of patients with at least a 1‐point improvement in the WGGSAIS, the odds ratio for velusetrag compared with placebo was calculated, with 95% confidence intervals (CIs) calculated using a logistic model for the end‐of‐treatment time point. A Fisher's exact test was applied to compare pairs of observations between treatments. For categorical variables (the number of pseudo‐obstructive episodes and the change in stool consistency), the Fisher's exact test was used to compare the distribution between the categories (0, 1, 2, or > 2 for the number of pseudo‐obstructive episodes; and constipation, normal, or diarrhea for stool consistency) at the end of treatment, and *p* values were calculated.

## 3 Results

### 3.1 Disposition, Demographics, and Characteristics

**FIGURE 1:**
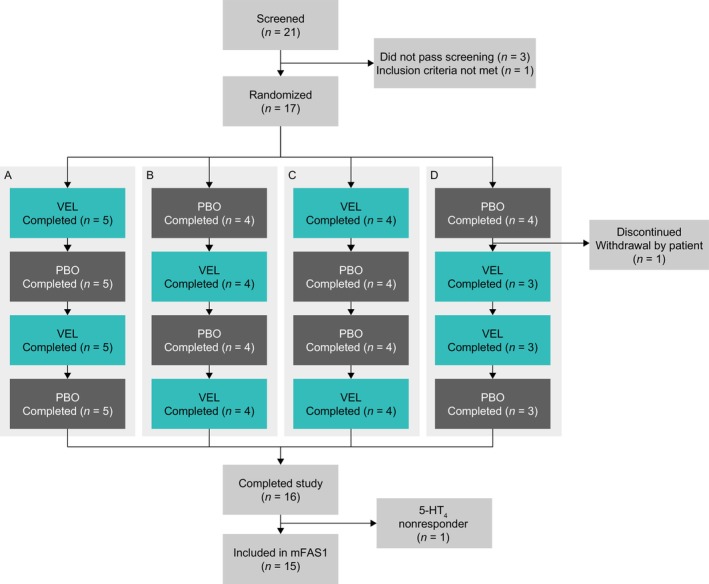
Patient disposition. Efficacy analyses were performed on the mFAS1 population (*n* = 15), which included all randomized and treated patients who were either responders or naive to previous treatment with a 5‐HT_4_ receptor agonist and who had at least one valid measurement for the primary endpoint during a VEL treatment period and a PBO treatment period within the same cycle. Safety analyses were performed on the safety analysis set population (*n* = 17), which included all patients who received at least one dose of VEL or PBO. 5‐HT_4_, 5‐hydroxytryptamine type 4; mFAS1, modified Full Analysis Set 1; PBO, placebo; VEL, velusetrag.

Between December 2021 and October 2022 (inclusive), 17 patients were randomized and received at least one study treatment from three of the planned four European hospital sites (two sites in Italy and one in Spain); 16 patients completed the study (Figure 1). Of the 17 randomized patients, two were excluded from the mFAS1 population used for efficacy analyses: one patient from the treatment sequence velusetrag–placebo–velusetrag–placebo who was a nonresponder to a previous 5‐HT_4_ receptor agonist, and one patient from the treatment sequence placebo–velusetrag–velusetrag–placebo who discontinued the study during the first treatment period owing to withdrawal by the patient. Baseline demographics and characteristics are shown in Table 1. Overall, most patients were female (80.00%), and the mean (standard deviation [SD]) age was 56.7 (10.65) years. All patients had a diagnosis of idiopathic CIPO, with a mean (SD) time from diagnosis of 8.02 (6.66) years. The mean (SD) number of pseudo‐obstructive episodes in the previous 6 months was 1.8 (2.34), ranging from 0 to 6, and the mean (SD) number of CIPO‐related hospitalizations in the previous 6 months was 0.3 (0.62), ranging from 0 to 2. At least one concomitant disease associated with CIPO was reported in 10 patients (66.67%). The most frequently reported concomitant diseases associated with CIPO (in ≥ 20% of patients) were gastrointestinal disorders (53.33%), musculoskeletal and connective tissue disorders (33.33%), and renal and urinary disorders (20.00%). Ten patients were responders to previous 5‐HT_4_ receptor agonist treatment, and five patients were naive to 5‐HT_4_ receptor agonist treatment (Table 1).

**TABLE 1:** Baseline demographics and characteristics.

	VEL–PBO–VEL–PBO	PBO–VEL–PBO–VEL	VEL–PBO–PBO–VEL	PBO–VEL–VEL–PBO	Total
	(*n* = 4)	(*n* = 4)	(*n* = 4)	(*n* = 3)	(*N* = 15)
Female, *n* (%)	4 (100)	2 (50.00)	3 (75.00)	3 (100)	12 (80.00)
Age, years, mean (SD)	51.5 (6.61)	59.3 (13.35)	57.5 (9.68)	59.3 (15.57)	56.7 (10.65)
Idiopathic CIPO diagnosis, *n* (%)	4 (100)	4 (100)	4 (100)	3 (100)	15 (100)
Time from CIPO diagnosis, years, mean (SD)	13.0 (6.28)	9.2 (8.59)	5.0 (3.77)	3.8 (4.74)	8.0 (6.66)
Number of pseudo‐obstructive episodes in the previous 6 months, mean (SD)	2.0 (2.83)	0.5 (1.00)	2.3 (2.63)	2.7 (3.06)	1.8 (2.34)
Number of CIPO‐related hospitalizations in the previous 6 months, mean (SD)	0	0.3 (0.50)	0.5 (0.58)	0.7 (1.15)	0.3 (0.62)
Responder status to 5‐HT_4_ receptor agonist treatment, *n* (%)					
Naive	0	1 (25.00)	2 (50.00)	2 (66.67)	5 (33.33)
Prior responder	4 (100)	3 (75.00)	2 (50.00)	1 (33.33)	10 (66.67)
Prior 5‐HT_4_ receptor agonist received, *n* (%)					
Prucalopride succinate	3 (75.00)	1 (25.00)	0	1 (33.33)	5 (33.33)
Prucalopride	1 (25.00)	1 (25.00)	2 (50.00)	0	4 (26.67)
Cisapride	1 (25.00)	1 (25.00)	0	0	2 (13.33)
Prior surgery related to CIPO, *n* (%)	3 (75.00)	3 (75.00)	1 (25.00)	2 (66.67)	9 (60.00)
Concomitant disease associated with CIPO, *n* (%)	3 (75.00)	2 (50.00)	3 (75.00)	2 (66.67)	10 (66.67)
Gastrointestinal disorders,^a^ *n* (%)	2 (50.00)	2 (50.00)	3 (75.00)	1 (33.33)	8 (53.33)
Musculoskeletal and connective tissue disorders,^b^ *n* (%)	2 (50.00)	1 (25.00)	1 (25.00)	1 (33.33)	5 (33.33)
Renal and urinary disorders,^c^ *n* (%)	1 (25.00)	0	1 (25.00)	1 (33.33)	3 (20.00)
Psychiatric disorders,^d^ *n* (%)	0	0	1 (25.00)	1 (33.33)	2 (13.33)
Blood and lymphatic system disorders,^e^ *n* (%)	1 (25.00)	0	0	0	1 (6.67)
Congenital, familial, and genetic disorders,^f^ *n* (%)	0	0	0	1 (33.33)	1 (6.67)
Endocrine disorders,^g^ *n* (%)	1 (25.00)	0	0	0	1 (6.67)
Immune system disorders,^h^ *n* (%)	0	0	1 (25.00)	0	1 (6.67)
Infections and infestations,^i^ *n* (%)	0	0	1 (25.00)	0	1 (6.67)
Metabolism and nutrition disorders,^j^ *n* (%)	0	0	1 (25.00)	0	1 (6.67)
Nervous system disorders,^k^ *n* (%)	0	0	1 (25.00)	0	1 (6.67)

*Note:* Data are reported from the mFAS1 population, which included all randomized and treated patients who were either responders or naive to previous treatment with a 5‐HT_4_ receptor agonist and who had at least one valid measurement for the primary endpoint during a VEL treatment period and a PBO treatment period within the same cycle.

Abbreviations: 5‐HT_4_, 5‐hydroxytryptamine type 4; CIPO, chronic intestinal pseudo‐obstruction; mFAS1, modified Full Analysis Set 1; PBO, placebo; SD, standard deviation; VEL, velusetrag.

^a^ Abdominal pain (*n* = 3), gastric dilatation (*n* = 3), constipation (*n* = 2), abdominal distension (*n* = 1), anal fissure (*n* = 1), anal ulcer (*n* = 1), Barrett's esophagus (*n* = 1), celiac disease (*n* = 1), colitis (*n* = 1), diarrhea (*n* = 1), gastrointestinal tract mucosal pigmentation (*n* = 1), intestinal dilatation (*n* = 1), rectal prolapse (*n* = 1), and rectocele (*n* = 1).

^b^ Osteoporosis (*n* = 4), muscle atrophy (*n* = 1), and osteopenia (*n* = 1).

^c^ Neurogenic bladder (*n* = 2) and cystitis (*n* = 1).

^d^ Depression (*n* = 1) and mixed anxiety and depressive disorder (*n* = 1).

^e^ Iron deficiency anemia (*n* = 1).

^f^ Gastrointestinal malformation (*n* = 1).

^g^ Hyperaldosteronism (*n* = 1).

^h^ Atopy (*n* = 1).

^i^ Vaginal infection (*n* = 1).

^j^ Hyperinsulinism (*n* = 1).

^k^ Chronic inflammatory demyelinating polyradiculoneuropathy (*n* = 1).

### 3.2 Efficacy Outcomes

**FIGURE 2:**
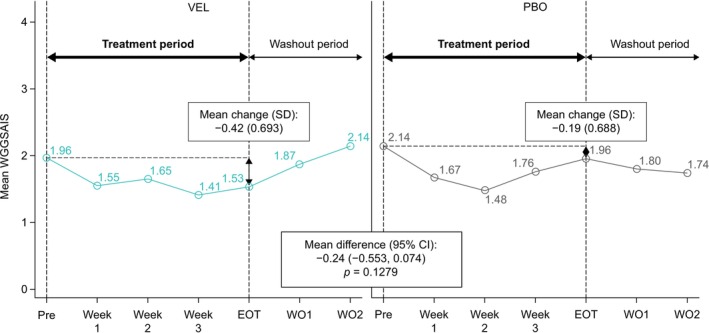
Primary endpoint: Mean WGGSAIS. Data are reported from the mFAS1 population, which included all randomized and treated patients who were either responders or naive to previous treatment with a 5‐HT_4_ receptor agonist and who had at least one valid measurement for the primary endpoint during a VEL treatment period and a PBO treatment period within the same cycle. Statistical analysis of the mean difference was performed using a paired *t*‐test. CI, confidence interval; EOT, end of treatment; mFAS1, modified Full Analysis Set 1; PBO, placebo; Pre, pretreatment; SD, standard deviation; VEL, velusetrag; WGGSAIS, weekly global gastrointestinal symptoms average index score; WO1, washout week 1; WO2, washout week 2.

The mFAS1 included 15 patients. Of the 30 potential evaluable velusetrag and placebo pairs for the primary endpoint analysis, there were 23 pairs with evaluable data (i.e., both pretreatment and end‐of‐treatment values were available for the velusetrag and placebo treatment periods within the same cycle). The mean (SD) change in the WGGSAIS from pretreatment to the end of treatment was −0.42 (0.693) for velusetrag and −0.19 (0.688) for placebo (between‐treatment difference: −0.24; 95% CI: −0.553, 0.074; *p* = 0.1279; Figure 2). In the sensitivity analysis with imputation of missing data (30 evaluable treatment pairs), the mean (SD) change in the WGGSAIS from pretreatment to the end of treatment was −0.59 (0.779) for velusetrag and −0.20 (0.736) for placebo (between‐treatment difference: −0.39; 95% CI: −0.741, −0.038; *p* = 0.0310). In total, 20 evaluable observed pairs were identified for the evaluation of at least a 1‐point improvement in the WGGSAIS. Of these, 30% of velusetrag observations (6/20) and 5% of placebo observations (1/20) achieved at least a 1‐point improvement in the WGGSAIS (odds ratio: 8.14; 95% CI: 0.88, 75.48; *p* = 0.0915).

There were 50% fewer pseudo‐obstructive episodes for velusetrag (four episodes) compared with placebo (eight episodes) from 30 evaluable pairs of observations. During velusetrag treatment, pseudo‐obstructive episodes occurred once in two pairs and twice in one pair, whereas during placebo treatment, seven pairs had one episode (*p* = 0.1455). One patient had a pseudo‐obstructive episode during the washout period after placebo treatment. There were no CIPO‐related hospitalizations during the study. Resolution of constipation was reported only with velusetrag treatment (Table 2). In 23 evaluable pairs, all four pairs with constipation before velusetrag treatment had normal stools at the end of velusetrag treatment (change from Bristol stool type 1 or 2 to type 3 or 4). In addition, the number of evaluable pairs with normal stools increased from three pairs before velusetrag treatment to six pairs after velusetrag treatment. In contrast, all three pairs with constipation before placebo treatment remained constipated at the end of placebo treatment. Two of the 17 pairs with diarrhea before placebo treatment were constipated at the end of placebo treatment (change in Bristol stool scale for velusetrag versus placebo, *p* = 0.0628; Table 2).

**TABLE 2:** Stool consistency.

*n*/*N* (%)	VEL				PBO			
	Bristol scale class pretreatment				Bristol scale class pretreatment			
	Constipation	Normal	Diarrhea	Total	Constipation	Normal	Diarrhea	Total
Overall	4/23 (17.39)	3/23 (13.04)	16/23 (69.57)	23/23 (100)	3/23 (13.04)	3/23 (13.04)	17/23 (73.91)	23/23 (100)
Bristol scale class at EOT								
Constipation	0	0	0	0	3/3 (100)	0	2/17 (11.76)	5/23 (21.74)
Normal	4/4 (100)	1/3 (33.33)	1/16 (6.25)	6/23 (26.09)	0	2/3 (66.67)	2/17 (11.76)	4/23 (17.39)
Diarrhea	0	2/3 (66.67)	15/16 (93.75)	17/23 (73.91)	0	1/3 (33.33)	13/17 (76.47)	14/23 (60.87)

*Note:* Data are reported from the mFAS1 population, which included all randomized and treated patients who were either responders or naive to previous treatment with a 5‐HT_4_ receptor agonist and who had at least one valid measurement for the primary endpoint during a VEL treatment period and a PBO treatment period within the same cycle.

Abbreviations: EOT, end of treatment; mFAS1, modified Full Analysis Set 1; PBO, placebo; VEL, velusetrag.

With velusetrag treatment (*n* = 7), the mean orocecal transit time decreased from screening to the end of the first treatment period (mean [SD]: screening, 169.3 [60.44] min; end of treatment, 162.9 [67.32] min; mean change [SD]: −6.4 [118.70] min). In contrast, the mean orocecal transit time increased from screening with placebo treatment (*n* = 6; mean [SD]: screening, 167.5 [61.79] min; end of treatment, 173.6 [52.58] min; mean change [SD]: 10.0 [55.05] min). The mean difference in the orocecal transit time with velusetrag compared with placebo was −16.4 min (95% CI: −133.0, 100.1; *p* = 0.7622). In the subgroup of patients who had not received antibiotics in the 14 days before the lactulose breath test, velusetrag treatment (*n* = 5) decreased the mean orocecal transit time from screening to the end of treatment (mean [SD]: screening, 195.0 [49.75] min; end of treatment, 132.0 [51.31] min; mean change [SD]: −63.0 [83.79] min). In contrast, placebo treatment (*n* = 4) increased the mean orocecal transit time in this subgroup (mean [SD]: screening, 187.5 [66.52] min; end of treatment, 206.3 [43.08] min; mean change [SD]: 18.8 [68.60] min). Among patients who had not received antibiotics in the 14 days before the lactulose breath test, the mean difference in the orocecal transit time with velusetrag compared with placebo was −81.8 min (95% CI: −204.9, 41.4; *p* = 0.1605).

### 3.3 Safety

The safety analysis set included all 17 patients who received at least one dose of velusetrag or placebo. The mean (SD) cumulative exposure was 8.25 (0.332) weeks for velusetrag and 8.01 (1.252) weeks for placebo. No deaths, serious TEAEs, TEAEs resulting in treatment interruption or discontinuation, or treatment‐related TEAEs (including cardiovascular treatment‐related TEAEs) were reported (Table S2). Seven velusetrag‐treated patients (41.18%) and 10 placebo‐treated patients (58.82%) reported at least one TEAE. A summary of TEAEs by system organ class and preferred term is shown in Table S3. During velusetrag treatment, mild TEAEs were reported in four patients (23.53%; with a total of 11 events), and moderate TEAEs were reported in three patients (17.65%; with a total of three events); no severe TEAEs were reported (Table S4). For placebo, mild TEAEs were reported in seven patients (41.18%; with a total of 17 events), and moderate TEAEs were reported in three patients (17.65%; with a total of four events); no severe TEAEs were reported (Table S4).

Three patients (17.65%) received concomitant CIPO treatments after starting velusetrag (magaldrate for acid‐related disorders; enemas and macrogol 3350/potassium chloride/sodium bicarbonate/sodium chloride for episodes of constipation; and pyridostigmine bromide as an intestinal prokinetic drug; each *n* = 1). One patient received concomitant CIPO medication after starting placebo (almagate for acid‐related disorders and hyoscine butylbromide for functional gastrointestinal disorders).

## 4 Discussion

This phase 2, proof‐of‐concept study examined the efficacy and safety of velusetrag in patients with idiopathic CIPO. Once‐daily, oral treatment with velusetrag 15 mg was associated with improvements in CIPO symptoms compared with placebo, with a mean decrease in the WGGSAIS from pretreatment to the end of treatment; although this difference did not achieve statistical significance. However, a prespecified sensitivity analysis, which used imputed data for missing evaluable treatment pairs, did demonstrate a significant decrease in the mean WGGSAIS with velusetrag from pretreatment to the end of treatment, providing further support for a treatment benefit of velusetrag over placebo. Although both velusetrag‐treated and placebo‐treated patients had decreases in the WGGSAIS in the first 2 weeks of treatment, this trend was maintained in the latter 2 weeks only with velusetrag; with placebo, the WGGSAIS increased to pretreatment levels. The improvement with velusetrag was modest, but this likely reflects the short treatment period.

Velusetrag also showed numerical improvements compared with placebo in secondary endpoints, including a greater proportion of patients with at least a 1‐point improvement in the WGGSAIS, fewer pseudo‐obstructive episodes (three pairs vs. seven pairs), and improvements in stool consistency. At the end of velusetrag treatment, there were no pairs with constipation compared with three pairs at the end of placebo treatment. A numerically lower orocecal transit time was observed with velusetrag compared with placebo, and the improvement with velusetrag compared with placebo was greater still when five patients who received antibiotics in the 14 days before the lactulose breath test were excluded from the analysis. It should be noted that the orocecal transit time reported following treatment with velusetrag (mean [SD]: 162.9 [67.32] min) was still higher compared with values previously reported for healthy individuals who had received lactulose solution (mean [SD]: 85.3 [42.8] min) [29]. Finally, a generally well‐tolerated safety profile was observed for velusetrag, and no treatment‐related cardiovascular TEAEs, serious TEAEs, or deaths were recorded. The TEAEs that were reported were generally mild and not thought to be related to treatment. Further studies with greater velusetrag exposure will facilitate a more comprehensive assessment of the safety profile of velusetrag.

Owing to their gastrointestinal prokinetic effects, 5‐HT_4_ receptor agonists have previously been evaluated in patients with CIPO. Cisapride, a first‐generation 5‐HT_4_ receptor agonist with low selectivity for 5‐HT_4_, has demonstrated some efficacy in CIPO [20]; however, it has since been withdrawn because of fatal cardiac arrhythmias and cardiovascular events [30]. Compared with first‐generation 5‐HT_4_ agonists, next‐generation 5‐HT_4_ agonists such as prucalopride have a higher specificity for intestinal 5‐HT_4_ receptors and have demonstrated stimulatory effects on gut motility without associated cardiotoxicity [30]. In a phase 2 study, four patients with CIPO received prucalopride and placebo in a crossover trial of four 12‐week treatment periods [21]. Prucalopride significantly improved bloating in four patients and abdominal pain in three patients compared with when these patients received placebo. However, prucalopride treatment was not associated with changes in stool frequency, stool consistency, or tolerance of oral feeding [21].

Velusetrag, a highly selective, next‐generation 5‐HT_4_ receptor agonist, has demonstrated gastrointestinal prokinetic activity in previous studies in animal models, healthy human volunteers, and patients with chronic constipation and diabetic or idiopathic gastroparesis [23, 24, 25, 26]. In a transgenic mouse model with gastrointestinal neuropathy, intestinal distension, and symptoms similar to CIPO, velusetrag reduced gut dilatation, improved intestinal damage in the distal small intestine and colon, and reduced neuronal loss compared with vehicle [31]. In healthy human volunteers, velusetrag treatment resulted in accelerated colonic transit compared with placebo [25]. When velusetrag efficacy was examined in a phase 2 study of patients with chronic idiopathic constipation, increased stool frequency, including the weekly frequency of spontaneous and complete spontaneous bowel movements, was observed compared with placebo [24].

Drug development for rare diseases is hindered by small patient populations, making it challenging to conduct clinical trials and gather sufficient data to demonstrate the efficacy and safety of new treatments. In addition, many rare diseases are poorly understood or recognized, leading to diagnostic delays. Thus, there remains a high unmet need for effective treatments for many rare diseases, such as CIPO, with a considerable burden and deleterious impact on patients' health‐related quality of life. Data from the current study add to the evidence base for velusetrag as a gastrointestinal prokinetic, demonstrating a positive effect of velusetrag treatment in patients with CIPO compared with placebo. This is important because patients with CIPO face substantial challenges. Impaired intestinal transit leads to chronic, debilitating symptoms, superimposed by episodic acute crises that may worsen over time. Together, these negatively affect health‐related quality of life, with a high degree of morbidity, intestinal failure, and early mortality [8, 9, 32, 33]. Children with intestinal pseudo‐obstruction have a high incidence of inpatient admission, high hospitalization costs, and a substantial healthcare burden [34]. These challenges are exacerbated by complications including malnutrition, requirement for parenteral nutrition, and central line‐associated bloodstream infections [34]. Investigating new treatment options that may reduce the frequency of pseudo‐obstructive crises is crucial to avoid unnecessary and potentially dangerous surgical procedures, to prolong the time until parenteral nutrition is required, and to improve survival [35].

### 4.1 Limitations

Given that CIPO is a rare disease, there was a limited number of patients available for recruitment into this study, making it challenging to demonstrate clear treatment efficacy. Moreover, the number of eligible patients with CIPO for the current study was further limited by the exclusion criteria, such as excluding those who were dependent on parenteral nutrition or who did not have adequate symptom reporting as stated in the protocol (Table S1). The treatment periods in this study were short, which may also have affected the ability to detect a sustained effect of velusetrag on the WGGSAIS compared with placebo. Additionally, the washout periods between treatment periods were kept relatively short (2 weeks) and were conservatively based on the documented half‐life of 17 and 30 h for velusetrag and its active metabolite, respectively. Despite this, it is possible that some pharmacodynamic effects of velusetrag from the most recent treatment period remained at the end of the washout period, although the reversal of symptomatic improvement during the washout periods following velusetrag treatment suggests that this effect is minimal. Furthermore, the presence of SIBO, which is relatively common in patients with CIPO [36], causes bacterial fermentation in the upper gastrointestinal tract, which could confound the results of the lactulose hydrogen breath test [37].

Furthermore, CIPO is a heterogeneous condition with multiple potential underlying pathogenetic mechanisms and comorbidities, which in turn may affect treatment outcomes. In addition, although patients with idiopathic CIPO or CIPO secondary to neurodegenerative or demyelinating disease were eligible for inclusion in this study, all enrolled patients had idiopathic CIPO. Furthermore, patients with idiopathic CIPO were not further stratified based on whether they had an underlying neuropathy or myopathy as this information was not collected. This, along with the small cohort size, prevented subgroup analyses.

Finally, there are no formal CIPO management guidelines, and limited numbers of clinical trials in patients with CIPO have been conducted, which can make designing an optimal trial challenging. Notably, there are currently no validated CIPO‐specific questionnaires to measure acute changes in disease severity; the choice of the WGGSAIS as the primary endpoint was based on expert opinion, which suggested that the most meaningful assessment of CIPO was to comprehensively assess the primary symptoms. Nonetheless, future assessment of velusetrag in CIPO with longer treatment durations would enable use of objective markers of disease activity such as artificial nutrition requirements and serum nutritional markers.

## 5 Conclusions

In this phase 2, proof‐of‐concept study, velusetrag treatment was associated with improved symptoms, a reduction in pseudo‐obstructive episodes, and normalized stool consistency compared with placebo, although without statistical significance. Velusetrag demonstrated a well‐tolerated safety profile.

## Author Contributions

Vincenzo Stanghellini: conceptualization and methodology. Carolina Malagelada, Roberto De Giorgio, Rosanna Francesca Cogliandro, Luis Alcalá‐González, Anna Costanzini, and Vincenzo Stanghellini: investigation. Rosanna Francesca Cogliandro, Luis Alcalá‐González, and Anna Costanzini: data curation, validation, and software. Rosanna Francesca Cogliandro, Luis Alcalá‐González, Anna Costanzini, and Elena Pasquali: formal analysis and visualization. Carolina Malagelada, Roberto De Giorgio, Valeria Scuderi, Sara Manzoni, Elena Pasquali, Jan Tack, and Vincenzo Stanghellini: data interpretation. All authors approved the final version of the article, including the authorship list.

## Funding

This study was sponsored by Alfasigma S.p.A.

## Disclosure

Guarantor of the article: Vincenzo Stanghellini.

## Ethics Statement

The study was approved by the Independent Ethics Committee or Institutional Review Board at the coordinating investigator's site (Comitato Etico Area Vasta Emilia Centro [CE—AVEC] at University of Bologna, IRCCS S. Orsola [no. 03901], reference number 652/2021/Farm/AOUBo) and at each individual study site (CE—AVEC at Arcispedale Sant'Anna di Ferrara [no. 03902], reference number 677/2021/Farm/AOUFe; Ethics Committee of the Hospital General Universitario Gregorio Marañón at Vall d'Hebron University Hospital [no. 03401], reference number 2021–000854‐24; and Ethics Committee Research at UZ Leuven [no. 03201], reference number S65763). The study was conducted in accordance with the ethical and scientific principles governing clinical research as set out in the Declaration of Helsinki, the guidelines on Good Clinical Practice, and applicable national and local laws and regulations.

## Consent

All participants provided written informed consent before participating in the study.

## Conflicts of Interest

C.M. has received consultancy fees from Alfasigma S.p.A. V.Sc. was an employee of Alfasigma S.p.A. at the time of the study. S.M. and E.P. are employees of Alfasigma S.p.A. J.T. has given scientific advice to Aclipse Therapeutics, Adare Pharma Solutions, Alfasigma, Clasado Biosciences, Danone, Dr. Falk Pharma, FitForMe, Ironwood Pharmaceuticals, Kyowa Kirin, Menarini Group, ProMed Pharma, Recordati, Takeda, Truvion Healthcare, Tsumura, and Zealand Pharma; has received research support from BIOHIT Healthcare, Kyowa Kirin, ProMed Pharma, Sofar, and Takeda; and has received speaker bureau fees from Abbott, Biocodex, Mayoly, Menarini Group, ProMed Pharma, Reckitt Benckiser, Schwabe Pharma, Takeda, Thai Meiji Pharmaceutical, and Truvion Healthcare. V.St. has received fees for advisory board participation and/or speaker fees for Alfasigma, Bayer, Bromatech, Dr. Falk Pharma, and GE Healthcare; and grants from Alfasigma S.p.A. All other authors declare that they have no relevant conflicts of interest to disclose.

## Supporting information


**Appendix S1:** nmo70246‐sup‐0001‐AppendixS1.docx.

## Data Availability

Due to the nature of the research and commercial restrictions, supporting data are not available.
