# Supplementary material for: Clinical Trial: Efficacy and Safety of Velusetrag in Chronic Intestinal Pseudo‐Obstruction: A Randomized, Phase 2, Placebo‐Controlled, Crossover, Multiple (n = 1), Proof‐of‐Concept Study
Source: Neurogastroenterol Motil. 2026 Jan 19;38(1):e70246. doi: 10.1111/nmo.70246 (PMC12814924; doi:10.1111/nmo.70246)
Supplement: Supplementary file 1 — Appendix S1: nmo70246‐sup‐0001‐AppendixS1.docx. [file NMO-38-e70246-s001.docx]

# Supporting Information

Clinical Trial: Efficacy and Safety of Velusetrag in Chronic Intestinal Pseudo-Obstruction: A Randomised, Phase 2, Placebo-Controlled, Crossover, Multiple (*n* = 1), Proof-of-Concept Study

Carolina Malagelada, Roberto De Giorgio, Rosanna Francesca Cogliandro, Luis Alcalá-González, Anna Costanzini, Valeria Scuderi, Sara Manzoni, Elena Pasquali, Jan Tack, Vincenzo Stanghellini

## Plain Language Summary

Chronic intestinal pseudo-obstruction (CIPO) is a serious condition where the gut cannot move food properly through the intestines, as if there is a blockage. Velusetrag is a medicine that stimulates gut movement, but it has not been assessed in CIPO. This study investigated whether velusetrag can be used to treat CIPO.

The study included 17 people with CIPO aged 18–80 years old. They received velusetrag and placebo (a tablet that looked like velusetrag but had no medicine in it) in a random order. The researchers wanted to know:

- Whether velusetrag improved CIPO symptoms like abdominal pain, bloating and vomiting, compared with placebo (measured using a symptom score).
- If there were any safety concerns of using velusetrag in people with CIPO.

Out of 17 patients, 15 patients had the necessary data for analysis. When patients received velusetrag, the symptom score decreased compared with the start of the study, indicating improvements in symptoms. When patients received placebo, the symptom score decreased but by less than it did when patients received velusetrag. However, although patients receiving velusetrag generally had greater improvement in symptoms than when they received placebo, the difference was not statistically significant (possibly due to the small number of patients). No deaths or serious medical problems (adverse events) related to the treatment were reported, and velusetrag was generally well tolerated.

Velusetrag may help to improve symptoms in people with CIPO. It was well tolerated with no serious adverse events. Further studies with more patients are needed to confirm these findings.

## TABLE S1 | Detailed inclusion and exclusion criteria.

| **Inclusion criteria** |
| --- |
| Male or female aged 18–80 years  History of CIPO, or CIPO secondary to neurodegenerative or demyelinating disease  Estimated oral daily caloric intake of ≥ 30% of the daily age- and sex-recommended caloric intake (stage 0–2 of the artificial food need scale)^a^  At least two out of four CIPO gastrointestinal symptoms (abdominal pain, bloating, nausea and vomiting), each with a score of ≥ 3 (0–4 scale), recorded in the gastrointestinal symptom questionnaire on day −1  Accepting and legally capable of providing free and informed consent to all procedures included in the protocol  All sexually active male participants who were partners to women of childbearing potential must have used a condom during intercourse until the ninetieth day after the end of the entire study  All female participants must have been of non-childbearing potential (post-menopausal [≥ 2 years without spontaneous menses], surgically sterile [bilateral tubal occlusion or hysterectomy] or had ablation of both ovaries) or of childbearing potential with a negative pregnancy test result at screening and randomisation and agreed to use a highly effective method of contraception (i.e., with a failure rate of < 1% per year) until the end of the entire study |
| **Exclusion criteria** |

| Primary CIPO, or CIPO secondary to other known endocrine/metabolic, autoimmune diseases and neurologic conditions other than neurodegenerative or demyelinating diseases  Conditions characterised by mechanical intestinal obstruction  Nasogastric tube, gastrostomy tube or jejunostomy feeding tube in place at randomisation or planned throughout the duration of the study, or artificial food need scale stage 3^a^  Presence of untreated thyroid dysfunction or known thyroid dysfunction not well controlled by treatment (e.g., patients with abnormal thyroid-stimulating hormone and, if available, abnormal triiodothyronine and thyroxin levels), deemed clinically significant by the investigator  History of diabetes at screening  Clinically significant ECG abnormalities (e.g., ST segment elevation or depression suggestive of ischaemia or partial or complete left bundle branch block) at screening and randomisation  Screening ECG with a Fridericia-corrected QT interval of > 450 ms in men or > 470 ms in women, or family history of sudden cardiac death  Required a low galactose diet  Hypersensitivity or documented intolerance to lactulose, lactose or any excipient of the lactulose preparation to be used for the lactulose breath test  History of sensitivity to velusetrag or any of the velusetrag or placebo excipients  Use of scopolamine or erythromycin in the 2 weeks before screening and/or planned throughout the duration of the study  Use of 5-HT_4_ receptor agonists (e.g., prucalopride, cisapride, clebopride or cinitapride) in the 5 days before randomisation and/or planned throughout the duration of the study  Use of opioids within 8 weeks from screening and/or planned throughout the duration of the study  Received strong CYP3A4 inhibitors (e.g., clarithromycin, indinavir, itraconazole, ketoconazole, nefazodone, nelfinavir, ritonavir, saquinavir, telithromycin or grapefruit juice), CYP3A4 inducers (e.g., rifampin, phenytoin, carbamazepine, phenobarbital or St John's wort), P-glycoprotein transporter inhibitors (e.g., captopril, carvedilol or diltiazem) or breast cancer resistance protein transporter inhibitors (e.g., curcumin, cyclosporine A or eltrombopag) in the 2 weeks before screening and/or planned throughout the duration of the study  Current swab-positive or suspected (under investigation) COVID-19  Cancer (excluding non-melanoma skin cancer) and/or need for any anti-cancer treatment (including radiotherapy) in the past 5 years  Severe kidney impairment (i.e., estimated glomerular filtration rate of < 30 mL/min)  Aspartate aminotransferase or alanine transaminase levels of > 2.5 times the upper limit of normal; bilirubin (unless deemed to be due to Gilbert’s syndrome) or alkaline phosphatase of > 1.5 times the upper limit of normal  Severe hepatic impairment defined as Child–Pugh class C  History of any of the following cardiac disorders:   - - Torsade de pointes, ventricular tachycardia or ventricular fibrillation   - Previous myocardial infarction, unstable angina pectoris, acute coronary syndrome, coronary artery or cerebral revascularisation procedure or stroke in the previous 18 months   - Angina pectoris class 2–4 during the 12 months before screening   - Congestive heart failure NYHA class III–IV during the 18 months before screening   History of any alcohol or drug abuse or dependence in the past year (investigator’s judgement)  Any current significant health condition (e.g., cardiovascular, respiratory, renal, hepatic, neurologic, psychiatric, haematologic, oncologic, immune, muscle or joint) that in the investigator’s judgement could have:   - - Jeopardised the patient’s safe participation in the trial   - Made it unlikely that the patient would complete the study   - Made it unlikely that the patient would adhere to the study procedures (e.g., highly anticipated need for non-permitted treatments, significant disability or terminal illness)   Pregnant or breastfeeding women  Use of any experimental drug in the 12 weeks before screening |
| --- |

^a^Artificial food need scale for patients with CIPO: stage 0 (free oral), stage 1 (modified oral in the absence of non-oral integration), stage 2 (oral with the need for non-oral integration) and stage 3 (full non-oral).

Abbreviations: CIPO, chronic intestinal pseudo-obstruction; CYP3A4, cytochrome P450-isozyme 3A4; ECG, electrocardiogram; NHYA, New York Heart Association.

**TABLE S2** | Summary of TEAEs.

|  | **VEL (*n*= 17)** | | **PBO (*n*= 17)** | |
| --- | --- | --- | --- | --- |
|  | ***n* (%)** | **Number of events** | ***n* (%)** | **Number of events** |
| **Any TEAEs** | 7 (41.18) | 29 | 10 (58.82) | 38 |
| **Treatment-related TEAEs** | 0 | 0 | 0 | 0 |
| **Any serious TEAEs** | 0 | 0 | 0 | 0 |
| **Treatment-related serious TEAEs** | 0 | 0 | 0 | 0 |
| **TEAEs resulting in treatment interruption or discontinuation** | 0 | 0 | 0 | 0 |
| **TEAEs resulting in death** | 0 | 0 | 0 | 0 |

*Note:* *n* is the number of patients with an event. Data are reported from the safety analysis set, which included all patients who received at least one dose of investigational treatment and were analysed according to the actual treatment received. TEAEs were defined as adverse events starting on or after the first intake of treatment (i.e., the first intake in period 1). TEAEs were classified as VEL-emergent if the last treatment taken before the TEAE onset date was VEL, or as PBO-emergent if the last treatment taken before the TEAE onset date was PBO.

Abbreviations: PBO, placebo; TEAE, treatment-emergent adverse event; VEL, velusetrag.

**TABLE S3** | Summary of TEAEs by system organ class and preferred term.

|  | **VEL (*n*= 17)** | | **PBO (*n*= 17)** | |
| --- | --- | --- | --- | --- |
|  | ***n* (%)** | **Number of events** | ***n* (%)** | **Number of events** |
| **Blood and lymphatic system disorders** | 1 (5.88) | 1 | 0 | 0 |
| **Anaemia vitamin B12 deficiency** | 1 (5.88) | 1 | 0 | 0 |
| **Cardiac disorders** | 1 (5.88) | 2 | 1 (5.88) | 2 |
| **Palpitations** | 1 (5.88) | 2 | 0 | 0 |
| **Sinus bradycardia** | 0 | 0 | 1 (5.88) | 2 |
| **Ear and labyrinth disorders** | 1 (5.88) | 1 | 0 | 0 |
| **Ear pain** | 1 (5.88) | 1 | 0 | 0 |
| **Eye disorders** | 1 (5.88) | 5 | 1 (5.88) | 10 |
| **Eye irritation** | 1 (5.88) | 1 | 0 | 0 |
| **Eye pruritus** | 1 (5.88) | 2 | 1 (5.88) | 1 |
| **Eye swelling** | 1 (5.88) | 2 | 1 (5.88) | 5 |
| **Ocular hyperaemia** | 0 | 0 | 1 (5.88) | 4 |
| **Gastrointestinal disorders** | 2 (11.76) | 2 | 3 (17.65) | 4 |
| **Constipation** | 1 (5.88) | 1 | 1 (5.88) | 2 |
| **Diarrhoea** | 0 | 0 | 1 (5.88) | 1 |
| **Intestinal pseudo-obstruction** | 0 | 0 | 1 (5.88) | 1 |
| **Toothache** | 1 (5.88) | 1 | 0 | 0 |
| **General disorders and administration-site conditions** | 4 (23.53) | 5 | 3 (17.65) | 4 |
| **Influenza-like illness** | 2 (11.76) | 3 | 2 (11.76) | 2 |
| **Pyrexia** | 1 (5.88) | 1 | 1 (5.88) | 1 |
| **Fatigue** | 0 | 0 | 1 (5.88) | 1 |
| **Injection-site pruritus** | 1 (5.88) | 1 | 0 | 0 |
| **Infections and infestations** | 2 (11.76) | 4 | 1 (5.88) | 1 |
| **COVID-19** | 1 (5.88) | 1 | 0 | 0 |
| **Cystitis** | 1 (5.88) | 1 | 0 | 0 |
| **Influenza** | 0 | 0 | 1 (5.88) | 1 |
| **Urinary tract infection** | 2 (11.76) | 2 | 0 | 0 |
| **Injury, poisoning and procedural complications** | 0 | 0 | 1 (5.88) | 2 |
| **Sunburn** | 0 | 0 | 1 (5.88) | 2 |
| **Musculoskeletal and connective tissue disorders** | 2 (11.76) | 3 | 1 (5.88) | 5 |
| **Arthralgia** | 0 | 0 | 1 (5.88) | 1 |
| **Back pain** | 1 (5.88) | 1 | 0 | 0 |
| **Bone pain** | 0 | 0 | 1 (5.88) | 1 |
| **Muscle contracture** | 0 | 0 | 1 (5.88) | 1 |
| **Myalgia** | 1 (5.88) | 1 | 1 (5.88) | 1 |
| **Pain in extremity** | 1 (5.88) | 1 | 1 (5.88) | 1 |
| **Nervous system disorders** | 3 (17.65) | 4 | 1 (5.88) | 1 |
| **Headache** | 3 (17.65) | 4 | 1 (5.88) | 1 |
| **Psychiatric disorders** | 0 | 0 | 1 (5.88) | 1 |
| **Anxiety** | 0 | 0 | 1 (5.88) | 1 |
| **Renal and urinary disorders** | 0 | 0 | 1 (5.88) | 3 |
| **Dysuria** | 0 | 0 | 1 (5.88) | 1 |
| **Urinary incontinence** | 0 | 0 | 1 (5.88) | 1 |
| **Urinary retention** | 0 | 0 | 1 (5.88) | 1 |
| **Reproductive system and breast disorders** | 0 | 0 | 1 (5.88) | 1 |
| **Pelvic pain** | 0 | 0 | 1 (5.88) | 1 |
| **Respiratory, thoracic and mediastinal disorders** | 1 (5.88) | 1 | 1 (5.88) | 1 |
| **Catarrh** | 1 (5.88) | 1 | 0 | 0 |
| **Oropharyngeal pain** | 0 | 0 | 1 (5.88) | 1 |
| **Skin and subcutaneous tissue disorders** | 0 | 0 | 2 (11.76) | 2 |
| **Skin depigmentation** | 0 | 0 | 1 (5.88) | 1 |
| **Urticaria** | 0 | 0 | 1 (5.88) | 1 |
| **Vascular disorders** | 1 (5.88) | 1 | 1 (5.88) | 1 |
| **Hypertension** | 1 (5.88) | 1 | 1 (5.88) | 1 |

*Note:* *n* is the number of patients with an event. Data are reported from the safety analysis set, which included all patients who received at least one dose of investigational treatment, and were analysed according to the actual treatment received. TEAEs were defined as adverse events starting on or after the first intake of treatment (i.e., the first intake in period 1). TEAEs were classified as VEL-emergent if the last treatment taken before the TEAE onset date was VEL, or as PBO-emergent if the last treatment taken before the TEAE onset date was PBO. Patients who experienced more than one TEAE were counted only once in each row. Terms were coded using the MedDRA version 26.0.

Abbreviations: MedDRA, Medical Dictionary for Regulatory Activities; PBO, placebo; TEAE, treatment-emergent adverse event; VEL, velusetrag.

**TABLE S4** | Summary of TEAEs by severity.

|  | **VEL (*n*= 17)** | | | **PBO (*n*= 17)** | | |
| --- | --- | --- | --- | --- | --- | --- |
| ***n* (%)** | **Mild** | **Moderate** | **Severe** | **Mild** | **Moderate** | **Severe** |
| **Any TEAEs** | 4 (23.53) | 3 (17.65) | 0 | 7 (41.18) | 3 (17.65) | 0 |
| **MedDRA system organ class/preferred term** |  |  |  |  |  |  |
| **Blood and lymphatic system disorders** | 1 (5.88) | 0 | 0 | 0 | 0 | 0 |
| **Anaemia vitamin B12 deficiency** | 1 (5.88) | 0 | 0 | 0 | 0 | 0 |
| **Cardiac disorders** | 1 (5.88) | 0 | 0 | 1 (5.88) | 0 | 0 |
| **Palpitations** | 1 (5.88) | 0 | 0 | 0 | 0 | 0 |
| **Sinus bradycardia** | 0 | 0 | 0 | 1 (5.88) | 0 | 0 |
| **Ear and labyrinth disorders** | 1 (5.88) | 0 | 0 | 0 | 0 | 0 |
| **Ear pain** | 1 (5.88) | 0 | 0 | 0 | 0 | 0 |
| **Eye disorders** | 1 (5.88) | 0 | 0 | 1 (5.88) | 0 | 0 |
| **Eye irritation** | 1 (5.88) | 0 | 0 | 0 | 0 | 0 |
| **Eye pruritus** | 1 (5.88) | 0 | 0 | 1 (5.88) | 0 | 0 |
| **Eye swelling** | 1 (5.88) | 0 | 0 | 1 (5.88) | 0 | 0 |
| **Ocular hyperaemia** | 0 | 0 | 0 | 1 (5.88) | 0 | 0 |
| **Gastrointestinal disorders** | 2 (11.76) | 0 | 0 | 2 (11.76) | 1 (5.88) | 0 |
| **Constipation** | 1 (5.88) | 0 | 0 | 1 (5.88) | 0 | 0 |
| **Diarrhoea** | 0 | 0 | 0 | 1 (5.88) | 0 | 0 |
| **Worsening of intestinal pseudo-obstruction** | 0 | 0 | 0 | 0 | 1 (5.88) | 0 |
| **Toothache** | 1 (5.88) | 0 | 0 | 0 | 0 | 0 |
| **General disorders and administration-site conditions** | 4 (23.53) | 0 | 0 | 3 (17.65) | 0 | 0 |
| **Fatigue** | 0 | 0 | 0 | 1 (5.88) | 0 | 0 |
| **Influenza-like illness** | 2 (11.76) | 0 | 0 | 2 (11.76) | 0 | 0 |
| **Injection-site pruritus** | 1 (5.88) | 0 | 0 | 0 | 0 | 0 |
| **Pyrexia** | 1 (5.88) | 0 | 0 | 1 (5.88) | 0 | 0 |
| **Infections and infestations** | 1 (5.88) | 1 (5.88) | 0 | 1 (5.88) | 0 | 0 |
| **COVID-19** | 1 (5.88) | 0 | 0 | 0 | 0 | 0 |
| **Cystitis** | 1 (5.88) | 0 | 0 | 0 | 0 | 0 |
| **Influenza** | 0 | 0 | 0 | 1 (5.88) | 0 | 0 |
| **Urinary tract infection** | 1 (5.88) | 1 (5.88) | 0 | 0 | 0 | 0 |
| **Injury, poisoning and procedural complications** | 0 | 0 | 0 | 0 | 1 (5.88) | 0 |
| **Sunburn** | 0 | 0 | 0 | 0 | 1 (5.88) | 0 |
| **Musculoskeletal and connective tissue disorders** | 2 (11.76) | 0 | 0 | 1 (5.88) | 0 | 0 |
| **Arthralgia** | 0 | 0 | 0 | 1 (5.88) | 0 | 0 |
| **Back pain** | 1 (5.88) | 0 | 0 | 0 | 0 | 0 |
| **Bone pain** | 0 | 0 | 0 | 1 (5.88) | 0 | 0 |
| **Muscle contracture** | 0 | 0 | 0 | 1 (5.88) | 0 | 0 |
| **Myalgia** | 1 (5.88) | 0 | 0 | 1 (5.88) | 0 | 0 |
| **Pain in extremity** | 1 (5.88) | 0 | 0 | 1 (5.88) | 0 | 0 |
| **Nervous system disorders** | 1 (5.88) | 2 (11.76) | 0 | 1 (5.88) | 0 | 0 |
| **Headache** | 1 (5.88) | 2 (11.76) | 0 | 1 (5.88) | 0 | 0 |
| **Psychiatric disorders** | 0 | 0 | 0 | 1 (5.88) | 0 | 0 |
| **Anxiety** | 0 | 0 | 0 | 1 (5.88) | 0 | 0 |
| **Renal and urinary disorders** | 0 | 0 | 0 | 0 | 1 (5.88) | 0 |
| **Dysuria** | 0 | 0 | 0 | 1 (5.88) | 0 | 0 |
| **Urinary incontinence** | 0 | 0 | 0 | 1 (5.88) | 0 | 0 |
| **Urinary retention** | 0 | 0 | 0 | 0 | 1 (5.88) | 0 |
| **Reproductive system and breast disorders** | 0 | 0 | 0 | 1 (5.88) | 0 | 0 |
| **Pelvic pain** | 0 | 0 | 0 | 1 (5.88) | 0 | 0 |
| **Respiratory, thoracic and mediastinal disorders** | 1 (5.88) | 0 | 0 | 1 (5.88) | 0 | 0 |
| **Catarrh** | 1 (5.88) | 0 | 0 | 0 | 0 | 0 |
| **Oropharyngeal pain** | 0 | 0 | 0 | 1 (5.88) | 0 | 0 |
| **Skin and subcutaneous tissue disorders** | 0 | 0 | 0 | 2 (11.76) | 0 | 0 |
| **Skin depigmentation** | 0 | 0 | 0 | 1 (5.88) | 0 | 0 |
| **Urticaria** | 0 | 0 | 0 | 1 (5.88) | 0 | 0 |
| **Vascular disorders** | 1 (5.88) | 0 | 0 | 0 | 1 (5.88) | 0 |
| **Hypertension** | 1 (5.88) | 0 | 0 | 0 | 1 (5.88) | 0 |

*Note:* *n* is the number of patients with an event. Data are reported from the safety analysis set, which included all patients who received at least one dose of investigational treatment, and were analysed according to the actual treatment received. TEAEs were defined as adverse events starting on or after the first intake of treatment (i.e., the first intake in period 1). TEAEs were classified as VEL-emergent if the last treatment taken before the TEAE onset date was VEL, or as PBO-emergent if the last treatment taken before the TEAE onset date was PBO. Patients who experienced more than one TEAE were counted only once in each row. Terms were coded using the MedDRA version 26.0.

Abbreviations: MedDRA, Medical Dictionary for Regulatory Activities; PBO, placebo; TEAE, treatment-emergent adverse event; VEL, velusetrag.

**FIGURE S1** | Study design.


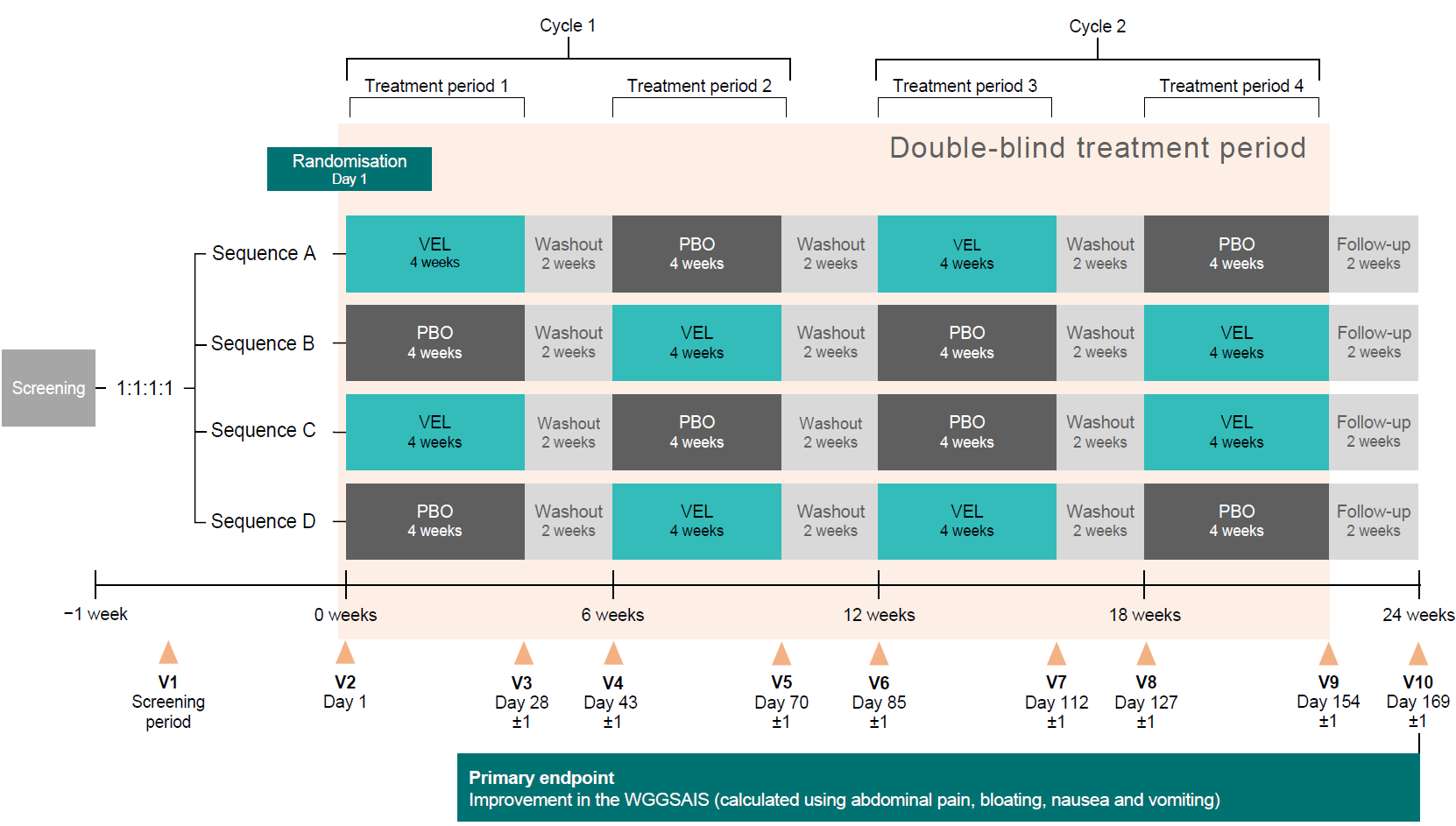


PBO, placebo; V, visit; VEL, velusetrag; WGGSAIS, weekly global gastrointestinal symptoms average index score.
